# Retraction: RTA408 alleviates retinal ganglion cells damage in mouse glaucoma by inhibiting excessive autophagy

**DOI:** 10.1371/journal.pone.0327995

**Published:** 2025-07-14

**Authors:** The PLOS One Editors

After this article [1] was published, concerns were raised regarding results presented in Figs 1, 2, 4, and 5.

Specifically,

- In Fig 4A, the Paracentral COH+RTA408 and Central COH+RTA408 panels appear similar.
- The following panels appear to partially overlap:
  - Fig 1D Paracentral COH+RTA408 and Central COH+RTA408
  - In Fig 2A:
    - Paracentral Control and Central Control,
    - Peripheral COH, Paracentral COH, and Central COH
    - Peripheral COH+TF and Paracentral C.O.H.+TF
    - Peripheral COH+RTA408 and Paracentral COH+RTA408
  - In Fig 4A:
    - Peripheral COH+RTA408, Paracentral COH+RTA408, and Central COH+RTA408,
    - Paracentral COH+RTA408+RaPa, and Central COH+RTA408+RaPa
    - Peripheral COH+RTA408+3MA and Paracentral COH+RTA408+3MA
  - In Fig 5A:
    - Paracentral COH and Central COH
    - Peripheral COH+RTA408+RaPa and Paracentral COH+RTA408+RaPa,
    - Peripheral COH+RTA408+3MA and Paracentral COH+RTA408+3MA,
- In Fig 5A, the Peripheral, Paracentral, and Central panels do not appear to match the corresponding whole retina panels for the COH and COH+RTA408+RaPa conditions.
- The Fig 5A Paracentral COH + RTA408 panel appears to be flipped horizontally compared to the corresponding whole retina COH + RTA408 panel.

The co-first author HQ stated that the overlaps between the Peripheral, Paracentral, and Central images identified in Figs 1D, 2A, 4A, and 5A are because the images are from adjacent regions on the same retina. They stated that these image overlaps do not affect the associated RGC quantifications in Figs 1E, 2B, 4B, and 5B as the quantification results in Figs 2B and 5B are derived from the whole retina images in Fig 2A and Fig 5A respectively, and the quantification results in Fig 1E and Fig 4B are derived from 40X images showing half the retina, not provided in the published article [1].

Regarding the Fig 4A Paracentral COH + RTA408 and Central COH + RTA408 panels, the corresponding author QW stated that an error occurred in the figure preparation and that the Central COH + RTA408 panel is incorrect.

Regarding the concerns for Fig 5A COH and COH + RTA408 + RaPa results not matching the whole retina images, the co-first author HQ stated that errors occurred in the figure preparation. They stated the Fig 5A COH whole retina panel is correct, but the COH Peripheral, Paracentral, and Central images are incorrect and that these incorrect panels correspond to the Fig 5A COH + RTA408 + RaPa group. PLOS therefore have concerns with the labeling, storage, and reliability of the data presented in this article [1].

A member of the *PLOS One* Editorial Board reviewed the concerns of the panel overlaps between the Peripheral, Paracentral, and Central images in Figs 1D, 2A, 4A, and 5A. They stated that these panel overlaps affect the interpretation of the results, as any differences between the retinal regions are difficult to determine. They stated that the article does not include a definition of the central, paracentral, and peripheral sections of the retina and that it is not clear whether the regions are defined similarly between the eyes examined. PLOS therefore remains concerned for the reliability of the retina region results.

Additionally, the *PLOS One* Editorial Board member raised concerns for the treatment of the animals, as the chronic ocular hypertension condition was established bilaterally in both eyes of the relevant mice. The use of both eyes may not align with internationally-accepted standards for the use of animals in research in place at the time of the article’s [1] publication, and bilateral treatment of the eyes does not appear to be justified in the article.

In light of the above concerns that question the reliability of the results and adherence to *PLOS One’s* publication policies, the *PLOS One* Editors retract this article.

HQ agreed with the retraction and stands by the article’s findings. QW did not respond to the final editorial decision. WC, GY, ML, LZ, BW, HH, JX, and ML either did not respond directly or could not be reached.

In addition to the above concerns, the *PLOS One* Editors noted errors in the labeling in the Supporting Information files in [1], which were resolved during editorial discussions with the co-first author HQ. Additionally, the underlying data provided in S2 File for the Control, COH, and COH + RTA408 results in Fig 1E are the same as in Fig 4B, and the underlying data for these results in Fig 2B are the same as in Fig 5B.
